# Pharmacokinetics, safety and tolerability of human recombinant alkaline phosphatase in healthy volunteers

**DOI:** 10.1186/cc14206

**Published:** 2015-03-16

**Authors:** E Peters, J Arend, R Tiessen, A Van Elsas, R Masereeuw, P Pickkers

**Affiliations:** 1 Radboudumc, Nijmegen, the Netherlands; 2 AM-Pharma, Bunnik, the Netherlands; 3 PRA Health Sciences, Zuidlaren, the Netherlands

## Introduction

Clinical trials showed renal protective effects of bovine intestinal alkaline phosphatase in critically ill patients with sepsis-associated acute kidney injury (AKI) [1,2]. Recently, human recombinant AP (recAP) was developed as a pharmacological attractive replacement. We conducted a phase I clinical trial to evaluate tolerability, safety and pharmacokinetics of recAP in healthy volunteers.

## Methods

In a randomized, double-blind, placebo-controlled phase I trial, healthy volunteers received via a 1-hour i.v. infusion a single dose of recAP (200, 500, 1,000 or 2,000 U/kg; *n *= 33) or multiple doses of recAP (500 or 1,000 U/kg; *n *= 18) on three consecutive days (*n *= 18). Serum recAP concentrations, AP activity levels and anti-drug antibodies were measured, and safety parameters were monitored.

## Results

RecAP administration resulted in a terminal elimination half-life and plasma clearance of 49 to 58 hours and 2.8 to 3.4 l/hour after single ascending doses, respectively, and 63 to 66 hours and 3.1 to 3.8 l/hour after multiple ascending doses. Peak recAP concentrations and AP activity levels were reached at the end of the 1-hour infusion and showed a rapid decline with about 10% of the maximum concentration remaining at 4 hours and less than 5% at 24 hours post start. Although the maximal concentration and total systemic exposure of recAP and AP activity increased slightly more than dose proportionally this is of no significance in the estimated therapeutic dose range. RecAP treatment was generally well tolerated and anti-drug antibodies could not be detected in serum.

## Conclusion

RecAP is characterized by a long serum terminal half-life, by stable serum AP levels and did not exert any safety concerns when administered to healthy volunteers. These results pave the way to investigate the potential of recAP as a new treatment option for sepsis-associated AKI in a phase II clinical trial, which will start at the end of 2014 [Clinical Trial Register:NCT02182440].

## References

[B1] Pickkers P Crit Care 2012 16 R14 10.1186/cc11159 22269279 PMC3396250

[B2] Heemskerk S Crit Care Med 2009 37 417 23 e1 10.1097/CCM.0b013e31819598af 19114895

